# Sex-determining region Y-box protein 3 induces epithelial-mesenchymal transition in osteosarcoma cells via transcriptional activation of Snail1

**DOI:** 10.1186/s13046-017-0515-3

**Published:** 2017-03-23

**Authors:** Manle Qiu, Daoyun Chen, Chaoyong Shen, Ji Shen, Huakun Zhao, Yaohua He

**Affiliations:** 1 Department of Sports Medicine, Shanghai Jiao Tong University Affiliated Sixth People’s Hospital, 600 Yishan Road, Shanghai, 200233 China; 2 Department of Gastrointestinal Surgery, West China Hospital, Sichuan University, Chengdu, 610041 Sichuan China

**Keywords:** Sex-determining region Y-box protein 3, Snail1, Transcriptional activation, Epithelial-mesenchymal transition, Osteosarcoma

## Abstract

**Background:**

The transcription factor sex-determining region Y-box protein 3 (SOX3) plays important roles in various types of cancer. However, its expression and function have not yet been elucidated in osteosarcoma (OS).

**Methods:**

The expression levels of SOX3 in OS tissues and OS cell lines were determined by quantitative real-time polymerase chain reaction (qRT-PCR) and Western blot analysis. The effects of SOX3 expression on OS cell biological traits were investigated by overexpressing and downregulating SOX3 protein. The expression of epithelial-mesenchymal transition (EMT) markers and transcription factors associated with EMT (EMT-TFs), were detected simultaneously. The mechanism underlying SOX3-mediated Snail1 expression was further investigated.

**Results:**

SOX3 was upregulated in human OS tissues. SOX3 overexpression promoted the EMT, migration and invasion in OS cells. The downregulation of SOX3 resulted in opposing effects. Furthermore, SOX3 upregulation enhanced the expression of the transcriptional repressor Snail1 by binding to its promoter region. Additionally, a positive correlation among the expression of SOX3, Snail1, and E-cadherin was demonstrated in human OS tissues.

**Conclusions:**

SOX3 promotes migration, invasiveness, and EMT in OS cells via transcriptional activation of Snail1 expression, suggesting that SOX3 is a novel regulator of EMT in OS and may serve as a therapeutic target for the treatment of OS metastasis.

## Background

Osteosarcoma (OS) is one of the most prevalent malignant bone tumors in childhood and adolescence. Similar to most other malignant tumors, OS is characterized by a high propensity for metastasis, which is the leading cause of death [1]. Despite several advancements in neoadjuvant chemotherapies and surgical methods in the treatment of OS, the 5-year survival of patients with metastatic disease is dismal (<20%) [2–4]. Therefore, it is necessary to urgently identify new targets or factors that govern metastasis and to develop novel therapeutic strategies for OS management.

Epithelial to mesenchymal transition (EMT), a developmental process in which epithelial cells lose polarity and develop a mesenchymal phenotype, plays an important role in the initiation of metastasis [5]. It is believed that EMT endows cancer cells with migratory and invasive properties, and induces cancer stem cell (CSC) properties [6]. The complex genetic changes during EMT are, at least in part, mediated by a number of specific transcription factors, such as Snail, Slug, Twist1, ZEB1, and ZEB2 [7–9]. Snail is overexpressed in various human solid tumors, including numerous types of carcinomas, as well as sarcomas, gliomas, neuroblastomas, and melanomas [10, 11]. Further, increased Snail1 expression in cancer cells promotes metastatic ability in vivo, as well as cell survival, angiogenesis, and chemoresistance in vitro [12–14]. In addition, exogenous overexpression of Snail1 increases the invasive and metastatic abilities of cancer cells through promoting the downregulation of E-cadherin and EMT [15]. However, the molecular mechanism underlying the upregulation of Snail1 in cancer cells remains unclear.

Sex-determining region Y-box protein 3 (SOX3) is a member of the Sox family of transcription factors that plays an important role in developmental processes, including neurogenesis [16], testis development [17], and chondrogenesis [18], among others [19, 20]. The expression of SOX3 decreases as development proceeds, and correlates with the cellular switch from proliferation to differentiation [21]. Recent studies have reported that SOX3 is upregulated in cancer tissues and may therefore play an oncogenic role in esophageal squamous cell carcinoma, ovarian cancer, and T-cell lymphomas [22–24]. Aberrant SOX3 expression also induces oncogenic transformation of chicken embryonic fibroblasts [25]. However, the function of SOX3 in OS progression has not yet been investigated.

In the present study, we first demonstrate that SOX3 expression is upregulated in human OS tissues and promotes metastatic potential both in vitro and in vivo. SOX3 also induces EMT via enhancing Snail1 expression in OS cells. Moreover, we reveal a positive correlation between SOX3 and Snail1 expression in OS samples.

## Methods

### Tissue specimens

mRNA and protein samples of human OS tissues (*n* = 42), the matched adjacent non-tumor samples (*n* = 42) and bone cysts (*n* = 28) were collected from patients who were admitted to Shanghai Jiao Tong University Affiliated Sixth People’s Hospital between 2009 and 2014. The adjacent non-tumor sample were resected within at least 5 cm of the tumor margin when the patients underwent definitive surgery. Pathological white slides of OS tissues and clinical information were obtained from 18 patients who were admitted to the cohort. Ethical approval for study was provided by the ethics committee of Shanghai Jiao Tong University Affiliated Sixth People’s Hospital. Written informed consent was obtained from all subjects or their guardians.

### Cell lines and cell culture

U2OS, SoSP-M, SoSP-9607, and MG-63 cells were purchased from the Cell Bank of China Academy of Sciences (Shanghai, China) and routinely checked for *Mycoplasma* contamination by Hoechst staining. U2OS, SoSP-M, SoSP-9607 were cultured in RPMI 1640 medium (Gibco, USA) supplemented with 10% fetal bovine serum (FBS) (Gibco, USA), 10 μg/ml streptomycin sulfate and 100 μg/ml penicillin G. MG-63 cell lines were cultured in high-glucose Dulbecco’s modified Eagle’s medium (DMEM; Gibco, USA) supplemented with 10% fetal bovine serum (FBS) (Gibco, USA), 10 μg/ml streptomycin sulfate and 100 μg/ml penicillin G. Cells were incubated at 37 °C in a humidified atmosphere containing 5% CO_2_.

### RNA isolation and real-time PCR analysis

Total RNA were extracted from fresh tissues and cells using TRIzol reagent (Invitrogen, CA, USA) according to the manufacturer’s protocol. Total RNA (500 ng) was reverse-transcribed into complementary DNA using the Reverse Transcription Reagent Kit (TaKaRa, Japan). Real-time PCR analysis was performed using the 7500 Real-Time PCR system (Applied Biosystems, USA) with a SYBR Green PCR Amplification Kit (TaKaRa). Primers are shown in Table 1. Each PCR analysis was performed in triplicate, and the results were normalized to β actin expression. The 2^-△△Ct^ method was used for data analysis.

**Table 1:** Primers used for the qRT-PCR analysis

genes	Sequence (5’-3’)	
	Forward primer	Reverse primer
SOX3	GACCTGTTCGAGAGAACTCATCA	CGGGAAGGGTAGGCTTATCAA
E-cadherin	CGAGAGCTACACGTTCACGG	GGGTGTCGAGGGAAAAATAGG
CK-18	GGCATCCAGAACGAGAAGGAG	ATTGTCCACAGTATTTGCGAAGA
N-cadherin	TCAGGCGTCTGTAGAGGCTT	ATGCACATCCTTCGATAAGACTG
Vimentin	GACGCCATCAACACCGAGTT	CTTTGTCGTTGGTTAGCTGGT
Snail1	AAGGCCTTCTCTAGGCCCT	CGCAGGTTGGAGCGGTCAG
Snail2	TTCGGACCCACACATTACCT	GCAGTGAGGGCAAGAAAAAG
Twist1	CAGCTACGCCTTCTCGGTCT	CTGTCCATTTTCTCCTTCTCTGGA
Zeb1	GATGATGAATGCGAGTCAGATGC	ACAGCAGTGTCTTGTTGTTGT
Zeb2	CAAGAGGCGCAAACAAGCC	GGTTGGCAATACCGTCATCC
Snail1 promoter	TCAGAAGCGCTCAGACCAC	TTATCTGCCACGCCCCTTT
Snail1 non-promoter	TGCTCATCTGGGACTCTGTC	GAGGAGAAGGACGAAGGAGC
β actin	TTGCCGACAGGATGCAGAAG	CAGCGAGGCCAGGATGGAGC

### Western blot analysis

Total proteins were extracted from fresh tissues and cells using RIPA Protein Lysis solution (Pierce, IL, USA) and quantified by the Bradford method. Prepared samples were electrophoresed by sodium dodecyl sulfate polyacrylamide gel electrophoresis and blotted onto a polyvinylidene fluoride membrane (Millipore, MA, USA) using the Tetra Handcast system (Bio-Rad, USA). The membrane was blocked for 1 h at room temperature or overnight at 4 °C in Tris-buffered saline with 0.05% Tween (TBST) containing 5% non-fat milk. Then it was incubated overnight at 4 °C with the appropriate primary antibody, and followed by the secondary antibody for 70 min at room temperature. The protein bands were detected and quantified using the Gene Gnome Syngene Bio Imaging System (Syngene, UK) with an electrochemiluminescence kit (Pierce, IL, USA).

The primary anti-SOX3 antibody was purchased from Abcam (MA, USA), and the anti-β actin antibody was purchased from Santa Cruz Biotechnology Inc. (CA, USA). Other primary antibodies (anti-E-cadherin, anti-CK-18, anti-N-cadherin, anti-vimentin, anti-Snail1, anti-Twist1, anti-Slug, anti-ZEB1, anti- ZEB2, anti-FLAG) were purchased from Cell Signaling Technology (MA, USA). Anti-mouse or anti-rabbit HRP-conjugated secondary antibodies were purchase from Santa Cruz Biotechnology Inc. (CA, USA).

### Lentivirus construction, Recombinant plasmid and siRNA transfection

Lentivirus construction of SOX3-shRNA, control shRNA, SOX3-overexpression, and control-overexpression were purchased from Genechem Company (Shanghai, China). SOX3-targeting shRNA were designed: shRNA-1: 5’- GCACATGAAGGAGTATCCGGA-3’; shRNA-2: 5’-GCCGTGCACATGAAGGAGTAT-3’; shRNA-3: 5’-GACGCTGCTCAAGAAAGATAA-3’; non-target control shRNA sequence: 5’-TTCTCCGAACGTGTCACGT-3’. Stable SOX3-knockdown and SOX3-upregulation were confirmed by quantitative real-time PCR and Western Blot analysis.

The plasmids expressed Snail1 and Snail1-siRNA were purchased from Genechem Company (Shanghai, China). The small interfering RNA (siRNA) sequence targeting Snail1 is as follows: 5’-GCGUGGGUUUUUGUAUCCA(dTdT)-3’. Cells were transfected with plasmid using Lipofectamine 2000 (Invitrogen) according to the manufacturer’s protocol.

### Luciferase reporter assays

The Snail1 promoter Luciferase reporter gene was generated from human genomic DNA corresponding to the sequence spanning −2,340 to +146 bp (relative to the transcriptional start site) of the 5’-flanking region of the human Snail1 gene. Various truncated reporter genes were generated as shown in Fig. 5. The constructs were confirmed by DNA sequencing. Cells were cultured in 24-well plates and transfected with 1ug reporter gene construct using Lipofectamine 2000 (Invitrogen) according to the manufacturer’s instructions. After 24 h of transfection, luciferase activities were measured using the Dual-Glo™ Luciferase Assay System kit (Promega, WI, USA). Luciferase intensities were normalized to the activity of Renilla luciferase.

### Chromatin immunoprecipitation assay

We constructed two FLAG-tagged SOX3 variants harboring a deletion of the HMG domain or the C-terminal transcriptional activation domain (SOX3-ΔHMG and SOX3-ΔCTD). After transfected with the plasmid for 48 h, OS cells were performed with Chromatin immunoprecipitation assay. OS cells were fixed with 1% formaldehyde and processed with the ChIP-IT Enzymatic kit (Active Motif, Carlsbad, CA) according to the manufacturer’s instruction. The resulting protein/DNA complexes were then immuno-precipitated overnight at 4 °C using anti-Flag antibody (Roche, CH), non-specific IgG antibodies (Santa Cruz Biotechnology Inc., CA, USA), respectively, with protein G magnetic beads. After eluted from beads and incubated with proteinase K, the precipitated DNA were amplified and quantified using PCRs. The primers for Snail1 promoter are shown in Table 1.

### Cell migration and matrigel invasion assays

Cell migration was evaluated using the wound-healing migration assays. Cells were cultured to form a tight cell monolayer and then wounded with 10 μL plastic pipette tip. The remaining cells were washed twice with culture medium to remove cell debris and cultured in normal serum-containing culture medium with the presence of Mitomycin C (Amresco) at the concentration of 10 μg/ml. The incubation is at 37 °C with humidified atmosphere containing 5% CO2. At the indicated times, migrating cells at the wound front were photographed. Migration capacity was quantitated by measuring the percent of open area in 3–5 randomly captured images.

Cell invasion assays were performed using 24-well Transwell chambers (8 μm pore size; Millipore) precoated with 100 ul Matrigel (1:7 dilution; BD Biosciences, San Jose, CA, USA). In total, 1 × 10^5^ cells were suspended in 100 μL serum-free medium with the presence of mitomycin C (10ug/ml) and were added to the upper chamber, and 600 μL DMEM that contained 10% FBS were placed in the lower chamber. After 24 h of incubation, Matrigel and the cells remaining in the upper chamber were removed by cotton swabs. Cells on the lower surface of the membrane were fixed in 4% paraformaldehyde and stained with crystal violet. Cells at × 200 magnification were counted and photographed. All experiments were performed in triplicate.

### In vivo lung metastasis model

The single-cell suspensions of 5×10^6^ OS cells/0.5 ml were injected into the tail vein of 5-week-old female Nude mice. The mice were sacrificed at 56 days after injection. The lungs were resected, and measured by fluorescent areas measured using the Image-Pro Plus software 6.0 (Media Cybernetics Inc., Silver Spring, MD, USA).

### Immunohistochemistry analysis

Immunohistochemical staining was performed according to the method of our previous studies [26]. Typically, after rehydration and antigen retrieval, the slides were incubated with primary antibodies against SOX3 (1:100, Abcam, MA, USA), Snail1(1:100, Cell Signaling Technology, MA, USA) and E-cadherin (1:100, Cell Signaling Technology, MA, USA) at 4 °C overnight, followed by HRP-conjugated secondary antibody at 37 °C for 30 min. Then the slides were stained with 3,3’-diaminobenzidine (DAB) and Mayer’s hematoxylin. Immunoreactivity Score was conducted by two independent observers, who were blinded to the patients’ information. Five high-power fields (magnification, 400×) were randomly selected. Based on the intensity of positive staining, immunoreactivity was categorized as follows: negative (−), weak (+−), moderate (+), strong (++). Negative and weak correspond to low expression, moderate and strong correspond to high expression.

### Statistical analysis

Statistical analysis was performed using SPSS16.0 statistical software (SPSS Inc., Chicago, IL, USA). Student’s *t*-tests were used to assess significant differences among study groups. The level of significance was set at *p* < 0.05. All experiments were performed at least in triplicate.

## Results

### SOX3 is upregulated in human OS tissues

**Fig. 1:**
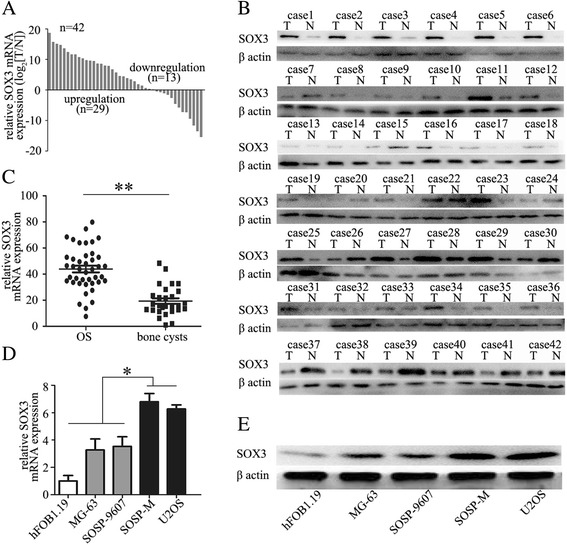
SOX3 is upregulated in human osteosarcoma tissues. **a**, **b** Relative SOX3 mRNA and protein levels were significantly increased in osteosarcoma tissues when compared with the adjacent non-tumor tissues (*n* = 42). **c** Relative SOX3 mRNA level was significantly higher in osteosarcoma tissues (*n* = 42) than that in bone cysts (*n* = 28). **d**, **e** SOX3 mRNA and protein levels in different OS cell lines. Data represent the mean ± SD of triplicate samples; *P < 0.05, **P < 0.01

We first determined the SOX3 mRNA and protein levels in 42 cases of human OS tissues and in adjacent non-tumor samples by qRT-PCR and Western blot analysis. SOX3 expression was increased in most OS samples relative to non-tumor samples (Fig. 1a, b). Next, the SOX3 mRNA level was assessed in OS tissues and bone cysts (Fig. 1c). SOX3 was overexpressed in most OS tissues compared with that in bone cysts. We further evaluated SOX3 expression in various OS cell lines with different metastatic potential (U2OS, SoSP-M, SoSP-9607, and MG-63) and the immortal normal osteoblast cell line, hFOB1.19. SOX3 was expressed at a relatively higher level in all four OS cell lines compared with hFOB1.19 (Fig. 1d), suggesting that SOX3 is upregulated in OS cell lines.

### SOX3 promotes OS cell migration and invasion

**Fig. 2:**
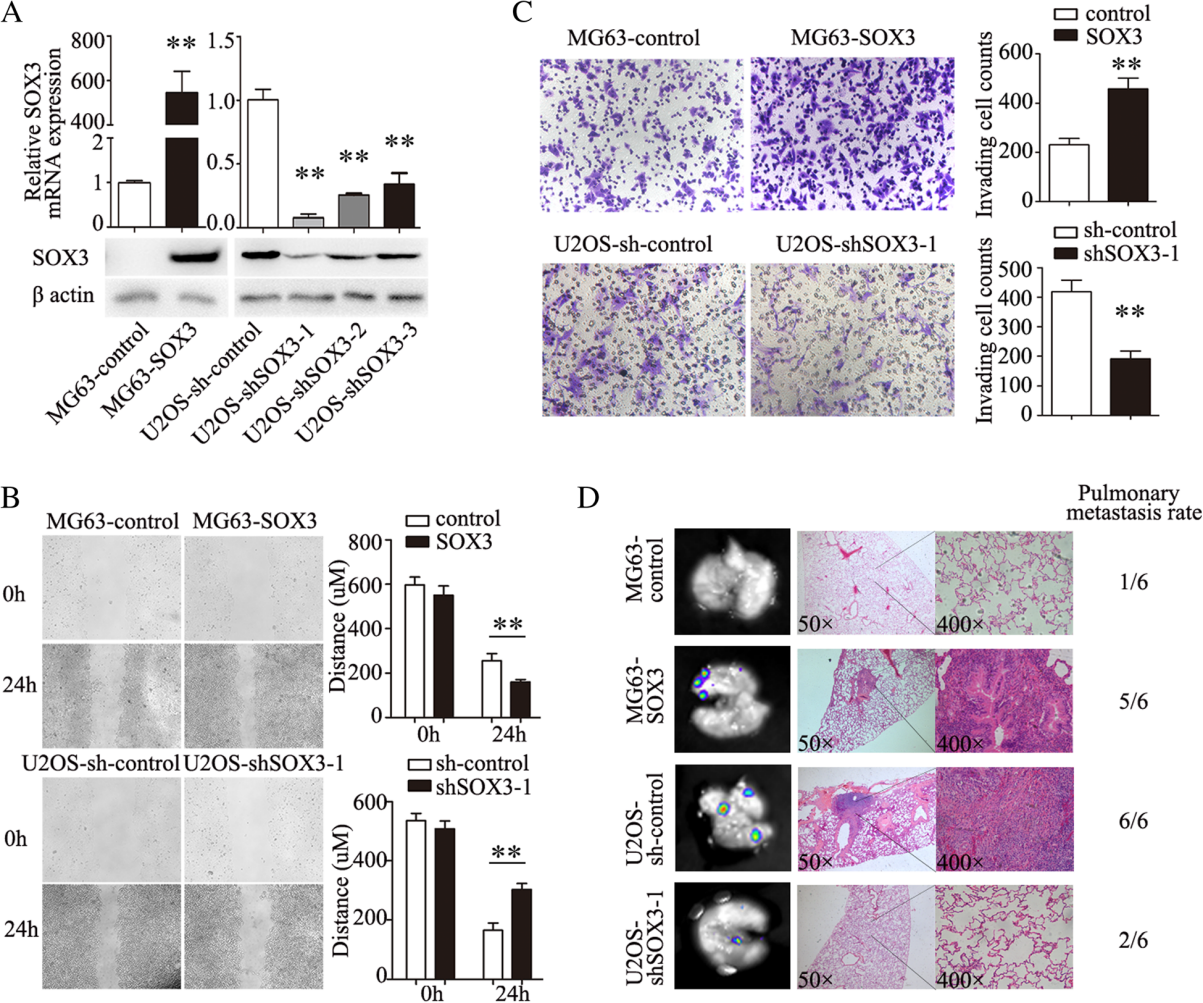
SOX3 promotes OS cell migration and invasion. **a** SOX3 overexpression in MG63 cells and successful knockdown of SOX3 in U2OS cells were confirmed by western blot and qRT-PCR. **b** Wound-healing migration assays and the distance quantification of wound closure. **c** The invasive OS cells and quantitation of invasive OS cell counts. **d** The lung metastatic foci were photographed. Representative views of lung tissue section are shown. The results are expressed as the mean ± SD of triplicate samples; * *P* < 0.05, **P < 0.01

To explore the role of SOX3 in OS progression, we examined the effects of SOX3 overexpression or knockdown on the migratory and invasive abilities of OS cells. The expression of SOX3 is relatively low in MG63 and high in U2OS cells. Therefore, we conducted stable SOX3 overexpression in MG63 cells (MG63-SOX3) and successful knockdown of SOX3 in U2OS cells (U2OS-sh-SOX3) (Fig. 2a). Wound-healing migration assays revealed that MG63-SOX3 cells had an increased rate of wound closure compared with the MG63-control, and that U2OS-sh-SOX3 cells had delayed wound closure compared with the U2OS-sh-control (Fig. 2b). Transwell® Matrigel® assays showed that the invasive MG63-SOX3 cell counts were significantly higher than that of the control cells and that the invasive U2OS-sh-SOX3 cell number was decreased compared with the control (Fig. 2c).

The lung is the most common site of OS metastasis. Therefore, we used a xenograft nude mouse model to examine the role of SOX3 expression in lung metastasis in vivo. We first injected MG63-control and MG63-SOX3 cells into nude mice via the tail vein. After 8 weeks, obvious lung metastases were detected in the lungs of recipient mice in the MG63-SOX3 group compared with the MG63-control group. We then injected U2OS-sh-control and U2OS-sh-SOX3 cells following the same protocol. As expected, knockdown of SOX3 significantly reduced lung metastasis in U2OS-sh-SOX3 cells compared with the U2OS-sh-control group (Fig. 2d). Therefore, SOX3 appears to play an oncogenic role in OS progression.

### SOX3 induces EMT and upregulates Snail1 expression in OS cells

**Fig. 3:**
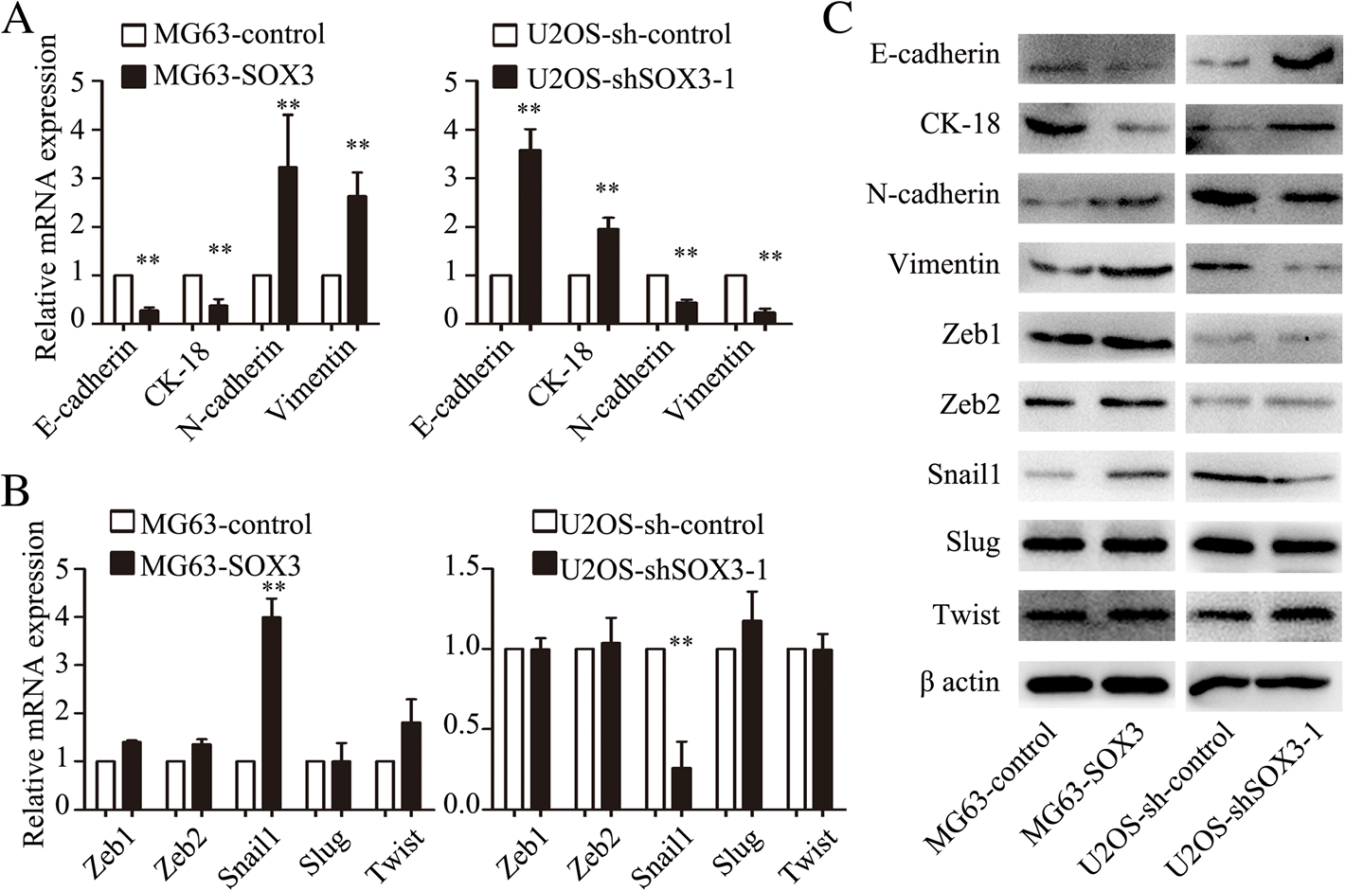
SOX3 induces EMT and upregulates Snail1 expression. **a** qRT-PCR shows changes of EMT marker expression affected by SOX3 upregulation and downregulation. **b** qRT-PCR shows changes of EMT-related transcription factors expression affected by SOX3 upregulation and downregulation. **c** western blot shows the protein expression of EMT markers and related transcription factors. The results are expressed as the mean ± SD of triplicate samples; * *P <* 0.05, **P < 0.01

EMT is the critical process and mechanism by which local invasion and distant metastasis are initiated. Overexpression of SOX3 in MG63 cells led to significantly higher levels of mesenchymal markers (N-cadherin and vimentin) and lower levels of epithelial markers (E-cadherin and Keratin 18) (Fig. 3a, c), whereas knockdown of SOX3 in U2OS cells caused increased expression of epithelial markers and decreased expression of mesenchymal markers (Fig. 3a, c). These data confirm that SOX3 induces EMT in OS cells.

To further define the mechanism by which SOX3 regulates EMT, we determined whether SOX3 modulates the expression of several known EMT-related transcription factors, including Zeb1, Zeb2, Snail1, Slug, and Twist1. We determined that the alterations in SOX3 expression led to significant changes in the expression of Snail1 (Fig. 3b, c), indicating that SOX3 may induce EMT by enhancing Snail1 expression.

### SOX3-promoted EMT is mediated by Snail1

**Fig. 4:**
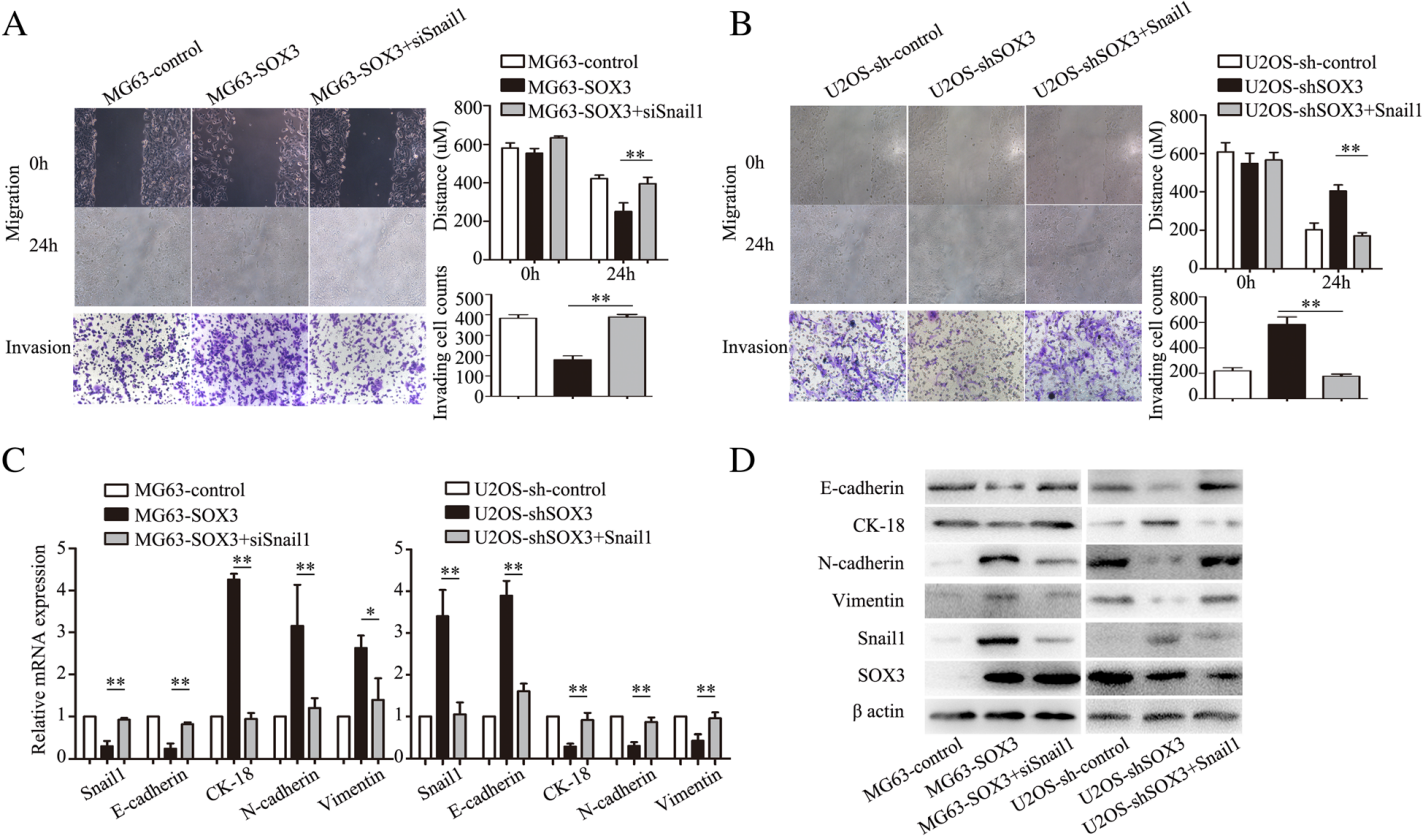
SOX3-induced EMT is mediated by Snail1 transcription factor. **a** siRNA against Snail1 abolished the increased cell ability of migration and invasion produced by SOX3 overexpression. **b** Snail1 plasmid (Snail1) significantly rescued the decreased cell migration and invasion produced by SOX3 knockdown. **c**, **d** the alterations of EMT markers by SOX3 were also reversed by siSnail1 or Snail1 plasmid. The results are expressed as the mean ± SD of triplicate samples; * *P <* 0.05, **P < 0.01

To confirm the involvement of Snail1 in the SOX3-induced EMT, siRNA targeting *Snail1* (siSnail1) was transfected into MG63-SOX3 cells. siSnail1 partially abolished the increased migratory and invasive abilities induced by SOX3 overexpression (Fig. 4a), whereas upregulation of Snail1 in U2OS-shSOX3 cells transfected with a Snail1 plasmid (Snail1) rescued the decreased cell migration and invasion induced by SOX3 knockdown (Fig. 4b). Additionally, qRT-PCR and Western blot analysis demonstrated that the alterations in EMT markers by SOX3 were reversed by siSnail1 or the Snail1 plasmid (Fig. 4c, d).

### SOX3 promotes Snail1 transcription via binding to its promoter region

**Fig. 5:**
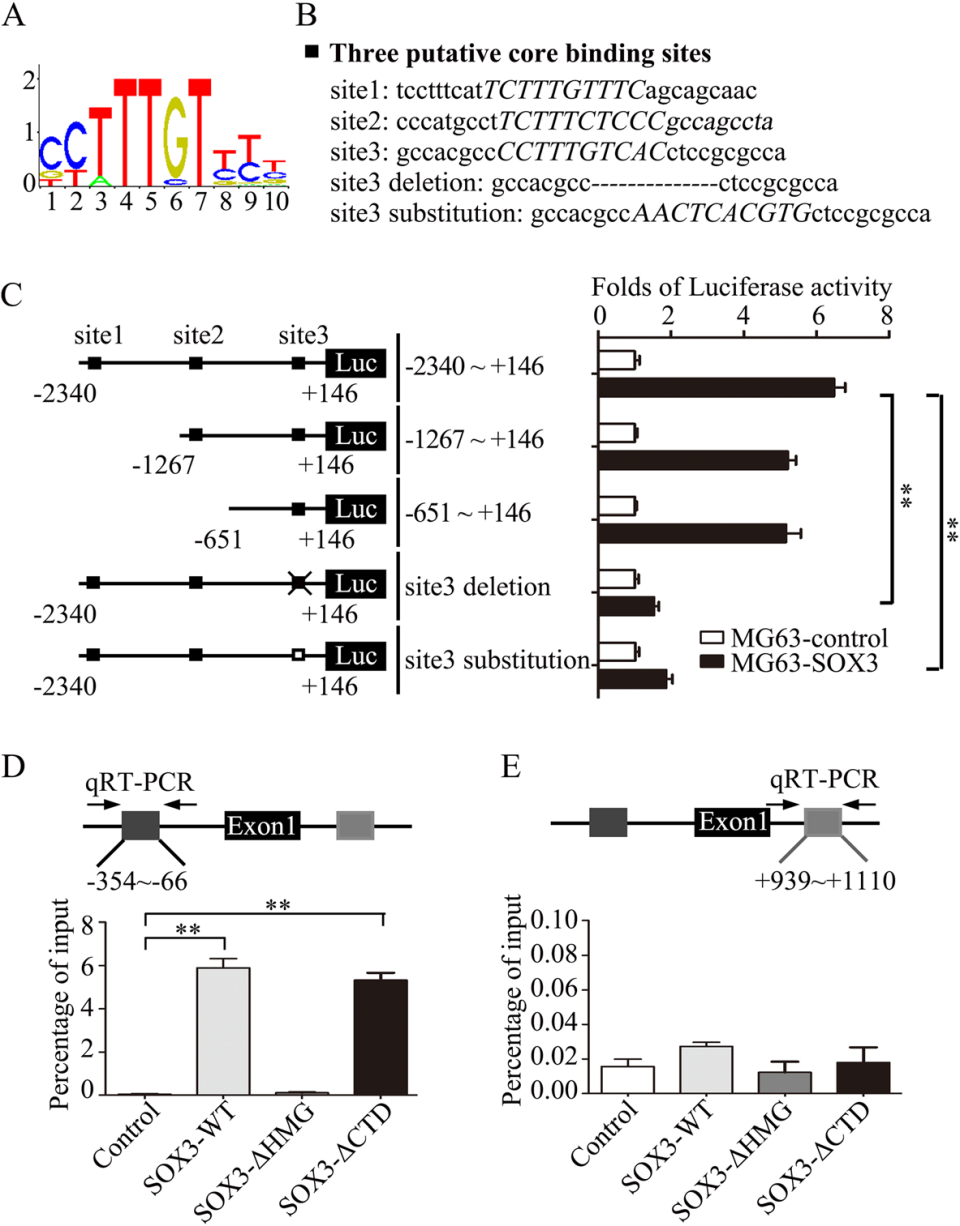
SOX3 promotes Snail1 transcription via binding to the promoter region. **a** Sequence logo of SOX3 from JASPAR database. **b** Three putative SOX3-binding sites in Snail1 promoter region. **c** Truncation analysis of the Snail1 reporters. Five kinds of reporters, including upstream regions from bp −2340, −1267, −651 to bp +146, and upstream regions with site 3 deletion or site 3 substitution, were analyzed for their activity. (**d**, **e**) FLAG-tagged SOX3 variants (SOX3-ΔHMG and SOX3-ΔCTD) were followed by chromatin immunoprecipitation (ChIP) assay. Precipitated DNAs were quantified by qRT-PCR for promoter and non-promoter regions of Snail1 gene. The results are expressed as the mean ± SD of triplicate samples; * *P <* 0.05, **P < 0.01

To explore the mechanism by which SOX3 regulates Snail1 expression in OS cells, we assessed the SOX3-dependent regulation of Snail1 mRNA transcription. The promoter region spanning −2,340 to +146 bp of Snail1 was used in a luciferase reporter assay. MG63-control and MG63-SOX3 cells were transfected with the reporter gene. SOX3 overexpression in MG63 cells promoted the reporter activity of Snail1 (Fig. 5c). Sequence analyses of the Snail1 promoter region showed three putative SOX3-binding sites (Fig. 5a, b). To determine the cis-regulatory elements of the Snail1 promoter in response to SOX3 regulation, various truncated reporter genes were constructed (Fig. 5c). SOX3 overexpression affected the transactivation of the shortest construct containing only the third binding site promoter sequence (−651 to +146 bp), while the deletion of sites 1 and 2 had no significant effect on SOX3-regulated Snail1 promoter activity. The deletion or substitution of site 3 showed notable inhibition of Snail1 promoter activity, suggesting that site 3 is the critical core binding site of SOX3 (Fig. 5c).

SOX3 contains a DNA-binding HMG domain and a C-terminal transcriptional activation domain. To determine the region required for its ability to transactivate Snail1 mRNA, we constructed two FLAG-tagged SOX3 variants harboring a deletion of the HMG domain or the C-terminal transcriptional activation domain (SOX3-ΔHMG and SOX3-ΔCTD, respectively) and performed a chromatin immunoprecipitation (ChIP) assay. SOX3-WT and SOX3-ΔCTD, but not SOX3-ΔHMG, pulled down the promoter region but not the non-promoter region of the *Snail1* gene (Fig. 5d, e). These data demonstrate that the HMG domain is critical for SOX3-activated Snail1 expression.

### SOX3 expression correlates with that of Snail1 and E-cadherin in human OS tissues

**Fig. 6:**
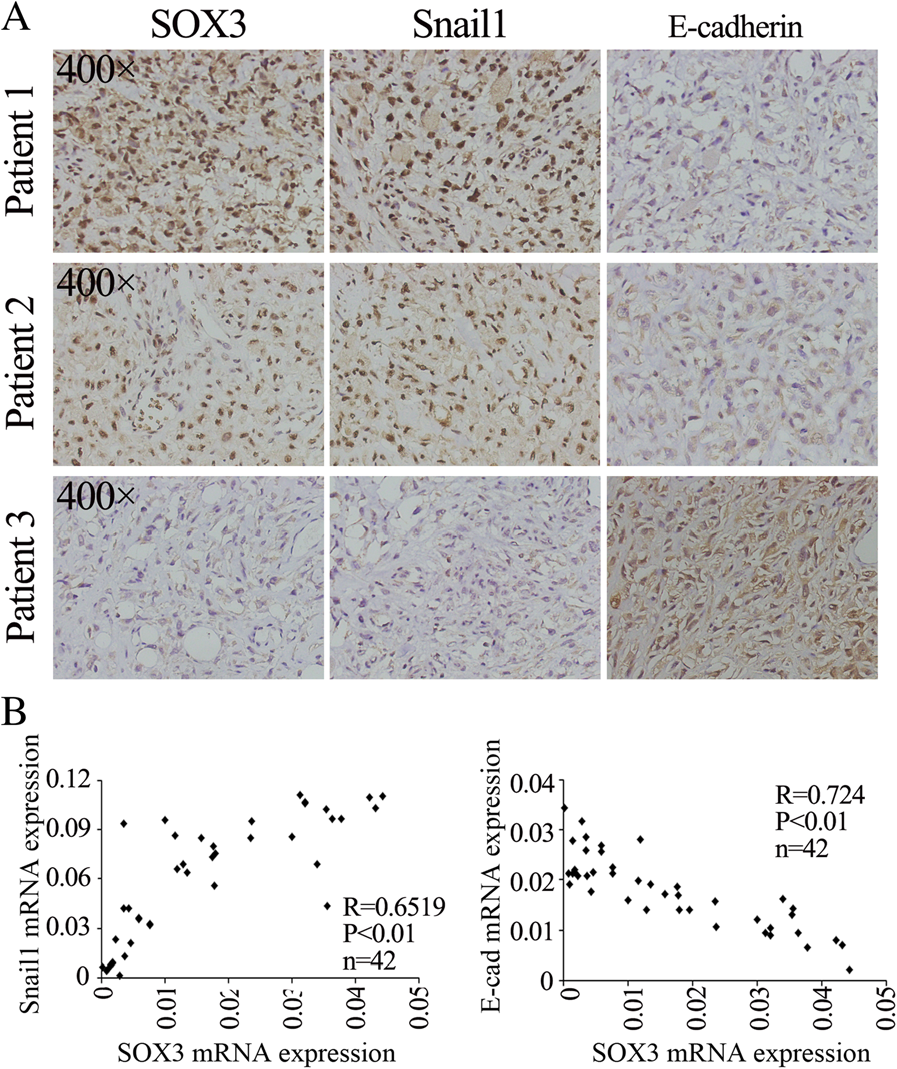
Correlation between the expression of SOX3 and Snail1, E-cadherin in patients with osteosarcoma. **a** The expression level of SOX3 by immunohistochemistry in 68 patients’ osteosarcoma tissues. **b** Correlation analysis between the expression of SOX3 and Snail1, E-cadherin

We next assessed the relationships between SOX3, Snail1, and E-cadherin expression by immunohistochemistry in 18 human OS tissues. Twelve OS tissues exhibited high expression of SOX3; 6 had low expression. Nine of 12 tumors with high SOX3 expression tended to have higher Snail1 levels and seven showed lower E-cadherin levels (Table 2). In contrast, 4 of 6 tumors with low SOX3 expression exhibited lower Snail1 expression and 5 exhibited higher E-cadherin expression (Table 2). These data suggest that SOX3 expression positively correlates with that of Snail1 and negatively correlates with E-cadherin (Fig. 6a). Additionally, correlation analysis between the mRNA expression of SOX3, Snail1, and E-cadherin in 42 OS tissues also show the similar results (Fig. 6b). We also analyzed the relationship between SOX3 and the clinical pathological characteristic (Table 2). However, there were no significant statistically.

**Table 2:** The relationship between SOX3 expression and clinicopathological features OS

Clinicopathological features		SOX3		*P* value
		Low	High	
Age (y)	≤20	3	8	0.494125
	>20	3	4	
Gender	Male	4	7	0.73244
	Female	2	5	
Grade	Grade1	2	2	0.637628
	Grade2	3	6	
	Grade3	1	4	
Metastasis	Yes	2	6	0.502335
	No	4	6	
5-year survival	Absent	1	5	0.288844
	Present	5	7	
Snail1	Low	4	3	0.087375
	High	2	9	
E-cadherin	Low	1	7	0.093533
	High	5	5

## Discussion

SOX3 is a member of the SOX family of transcription factors that plays important roles in the regulation of embryonic development and in the determination of cell fate [17, 19, 20]. Recent studies have reported that SOX3 is linked to various cancers, including esophageal squamous cell carcinoma, ovarian cancer, and T-cell lymphomas [22, 23, 25]. In the present study, we first demonstrated that SOX3 was expressed at a higher level in OS tissues compared with normal tissues. In vivo and in vitro experiments suggested that SOX3 promoted OS cell invasion and metastasis. Further analysis showed that SOX3 significantly induced EMT by promoting Snail1 expression. Moreover, a positive correlation between SOX3 and Snail1 expression was validated in OS samples. These data suggest that SOX3 plays a crucial role in OS progression.

Cancer has been proposed to have six fundamental hallmarks [27], which include self-sufficiency in growth factors, insensitivity to growth-inhibitory signals, evasion of apoptosis, limitless replicative potential, sustained angiogenesis, and the ability to invade and metastasize. The primary tumor is rarely fatal to the organism. It is the final hallmark of cancer, invasion and metastasis, which underlies its deadly progressive nature [27]. Metastatic spread is responsible for more than 90% of all cancer-related deaths, yet, despite this being well-known, metastasis in and of itself remains the most poorly understood component of cancer progression. OS is also characterized by a high propensity for metastasis (especially to the lung), which is the leading cause of death [1]. Our study revealed a crucial role for SOX3 in promoting OS invasion and metastasis both in vitro and in vivo, strongly suggesting that SOX3 may serve as a therapeutic target for patients with metastatic OS.

EMT is a key event in the tumor invasion and metastasis process that enables epithelial cells to acquire an invasive mesenchymal phenotype. EMT is characterized by a loss of intercellular adhesion (E-cadherin), downregulation of epithelial makers (cytokeratins), upregulation of mesenchymal markers (vimentin), acquisition of a spindle-like morphology, and an increase in motility, invasiveness, and metastatic capabilities [6]. In addition, the process known as “cadherin switching” (downregulation of E-cadherin and upregulation of mesenchymal cadherins such as N-cadherin [28]) and the accumulation of β-catenin have also been associated with EMT [29]. Previous studies have revealed the increased EMT characteristics in OS cells and demonstrated the involvement of EMT in OS invasiveness and migration [30–33]. In our study, the decreased invasive ability of the U2OS cell line induced by the depletion of SOX3 prompted us to investigate whether SOX3 is involved in EMT. As expected, SOX3 silencing upregulated epithelial markers, such as E-cadherin, and downregulated mesenchymal markers, such as N-cadherin and vimentin. Consistent with these data, ectopic expression of SOX3 in MG63 cells led to opposing results. This involvement of SOX3 in regulating EMT provided an explanation for the increased levels of SOX3 expression promoting OS cell invasiveness and metastasis.

EMT is a very complex process that is executed in response to pleiotropic signaling factors that include several transcriptional repressors, such as Snail1, Snail2, Zeb1, Zeb2, and Twist. The roles of these factors in promoting invasiveness and metastasis have been widely reported [34–38]. However, the upstream mechanism of these transcriptional repressors in cancer progression are still elusive. Previous studies revealed that SOX family of proteins play important role in cancer development [39–41]. For example, SOX5 promoted EMT process by regulation of Twist1 in hepatocellular carcinoma [41]. These literatures give us an implication that SOX3, as a member of SOX family, might play the potential role in regulating EMT-related transcriptional factors. Therefore, our study investigated the hypothesis and demonstrated that SOX3 enhanced Snail1 expression via binding to the site 3 region of the Snail1 promoter, thereby inducing EMT and promoting OS cell invasion and metastasis.

Previous studies have reported that SOX3 is correlated with the development of several types of cancer, including esophageal squamous cell carcinoma, ovarian cancer, and T-cell lymphomas, and induces oncogenic transformation of chicken embryonic fibroblasts [22, 23, 25]. These findings, together with our results showing that SOX3 is dramatically overexpressed in OS tissues and promotes OS cell invasion and metastasis, suggest that SOX3 may act as an oncogene. However, before defining SOX3 as an oncogene in OS progression, the relationship between SOX3 expression and the prognosis, pathology, and clinical phenotype of patients with OS should be investigated. Our studies showed that there was no significant relationship between SOX3 expression and the clinical pathological features in 18 patients with OS. This might be related to the limited sample size. The collection of a larger number of samples and clinical information is currently underway.

## Conclusion

In conclusion, the collective findings from our study show for the first time that SOX3 acts as a metastasis-associated gene in OS. The mechanistic link among SOX3, Snail1, and EMT indicates SOX3 as a potential therapeutic target for OS metastasis.

## Abbreviations

ChIP: Chromatin immunoprecipitation
CK-18: Keratin 18
CSC: Cancer stem cell
EMT: Epithelial-mesenchymal transition
EMT-TFs: Transcription factors associated with EMT
OS: Osteosarcoma
qRT-PCR: Quantitative real-time polymerase chain reaction
siSnail1: siRNA targeting *Snail1*
SOX3: Sex-determining region Y-box protein 3
